# Community-based post-stroke service provision and challenges: a national survey of managers and inter-disciplinary healthcare staff in Ireland

**DOI:** 10.1186/1472-6963-12-111

**Published:** 2012-05-06

**Authors:** Anne Hickey, Frances Horgan, Desmond O’Neill, Hannah McGee

**Affiliations:** 1 Department of Psychology, Division of Population Health Sciences, Royal College of Surgeons in Ireland, 123 St. Stephen's Green, Dublin 2, Ireland; 2 School of Physiotherapy, Royal College of Surgeons in Ireland, 123 St. Stephen's Green, Dublin 2, Ireland; 3 Centre for Ageing, Neuroscience and the Humanities, Trinity College, Dublin 2, Ireland

## Abstract

**Background:**

The extent of stroke-related disability typically becomes most apparent after patient discharge to the community. As part of the Irish National Audit of Stroke Care (INASC), a national survey of community-based allied health professionals and public health nurses was conducted. The aim was to document the challenges to service availability for patients with stroke in the community and to identify priorities for service improvement.

**Methods:**

The study was a cross-sectional tailored interview survey with key managerial and service delivery staff. As comprehensive listings of community-based health professionals involved in stroke care were not available, a cascade approach to information gathering was adopted. Representative regional managers for services incorporating stroke care (N = 7) and disciplinary allied health professional and public health nurse managers (N = 25) were interviewed (94% response rate).

**Results:**

Results indicated a lack of formal, structured community-based services for stroke, with no designated clinical posts for stroke care across disciplines nationally. There was significant regional variation in availability of allied health professionals. Considerable inequity was identified in patient access to stroke services, with greater access, where available, for older patients (≥ 65 years). The absence of a stroke strategy and stroke prevalence statistics were identified as significant impediments to service planning, alongside organisational barriers limiting the recruitment of additional allied health professional staff, and lack of sharing of discipline-specific information on patients.

**Conclusions:**

This study highlighted major gaps in the provision of inter-disciplinary team community-based services for people with stroke in one country. Where services existed, they were generic in nature, rarely inter-disciplinary in function and deficient in input from salient disciplines. Challenges to optimal care included the need for strategic planning; increased funding of healthcare staff; increased team resources and teamwork; and removal of service provision barriers based on age. There were notably many challenges beyond funding. Similar evaluations in other healthcare systems would serve to provide comparative lessons to serve to tackle this underserved aspect of care for patients with stroke and their families.

## Background

Approximately half of survivors of an acute stroke make a complete recovery [1]. Of the remaining 50%, approximately 30% will make an incomplete recovery, with the remaining 20% requiring assistance with at least one usual care activity. Physical disability and morbidity resulting from stroke pose a significant burden both at individual and societal level [2]. Social and psychological consequences include feelings of worthlessness, hopelessness and other depressive symptoms [3,4].

Returning to the community after acute hospitalisation for stroke can be difficult for the stroke patient, their primary carer and the extended family. The stroke patient has to assume increased responsibility for independent functioning in the absence of the supportive environment of the acute phase inpatient hospital setting. Patients frequently have persisting clinical problems including impaired upper and lower limb function, speech and cognitive problems, difficulties mobilising indoors, outdoors and on stairs, limited independence in self care, depression, and social inactivity [5]. Continuity of services is important but many reports describe unmet service and information needs of stroke patients and families following discharge from hospital [6,7]. No national study of the perspective of inter-disciplinary healthcare workers and managers of service provision for stroke in the community exists in the biomedical literature. A study of this type could provide valuable insights into barriers and enablers for the development of high-quality rehabilitation and support services in the community.

Service organisation can have an important effect on patient outcome [8,9]. The multidisciplinary team (MDT) is necessary to deliver comprehensive rehabilitation, whether in hospital or the community The early supported discharge (ESD) approach, which provides patients with a substantial part of their rehabilitation at home, has been shown in randomised controlled trials to reduce length of stay and deliver better long-term patient functional outcomes [10-14].

Evaluation of the benefits of community rehabilitation following stroke is limited and has been attributed to the lack of a clear definition on what constitutes the service, how it is organised, the level of specialisation, and duration of the service. Geddes and Chamberlain [15] evaluated six community services providing multidisciplinary community-based rehabilitation and found much variability in the target populations, and the timing and duration of interventions, thus making comparisons difficult. The significant medical, social, psychological, and economic ramifications of stroke, in conjunction with a projected rise in the number of stroke patients due to population ageing [16], highlight the need to ensure that adequate community services are available to ensure a seamless transfer after the acute hospital phase of treatment.

The aim of the current study was to document the availability of structures for supporting stroke care in the community following discharge from hospital. Available evidence has highlighted a number of deficiencies in community services for stroke in Ireland, including an absence of services such as early supported discharge and dedicated community stroke services, with ongoing unmet medical and rehabilitation needs for physiotherapy, occupational therapy and day care [17]. This study was one component of the Irish National Audit of Stroke Care (INASC) [18].

## Methods

Health services in Ireland are provided by the Health Services Executive (HSE), which is organised into four administrative geographic regions. These regions are further divided into 32 local health areas (managed by local health offices (LHO’s)), which deliver services at community level. As part of INASC, health managers involved in the care and management of stroke patients in the community following hospital discharge were surveyed. These included local health office (LHO) managers who were responsible at regional level for management and care of people with stroke, public health nurse managers and all allied health professional managers with potential involvement in stroke care in the community.

The survey was cross-sectional. As stroke care is one component of the job specification for many community-based health professionals, rather than a full job specification, comprehensive listings of staff involved in stroke care were not available for most relevant professional groups. Thus, since it was not possible to randomly survey staff, a cascade approach to information gathering was adopted. This involved overall regional managers identifying discipline managers at local health office level to the research team.

Across the 32 local health offices nationally, no managers had specifically identified responsibility for stroke services. Instead, in each region, the manager with regional responsibility for Services for Older People and the manager with regional responsibility for Services for People with Disabilities both had stroke care as part of their brief (N = 8). These regional managers were invited to participate in an interview and to nominate discipline-specific managers in their region with responsibility for stroke. The seven disciplines involved were public health nursing, physiotherapy, occupational therapy, speech and language therapy, nutrition and dietetics, social work and psychology. Community psychiatric nursing was not included since they have no involvement with stroke care in the community unless the person with stroke also has a diagnosed psychiatric condition.

A series of survey instruments was developed with similar themes but questions tailored to the specific professional group (health service versus discipline-specific managers) being surveyed. The eight health service managers were interviewed about stroke service planning and provision, including their role, in their local health area. The discipline-specific manager’s interview focussed on available services and on levels of staffing within their discipline for stroke service provision. All invitees were provided with interview schedules in advance to maximise the opportunity to collect or consult about information needed.

The study was approved by the Research Ethics Committee of the Royal College of Surgeons in Ireland (reference number: 185).

## Results

Of the 8 regional health service managers contacted, 7 agreed to take part: all 4 managers with responsibility for Services for People with Disability and 3 of 4 managers with responsibility for Services for Older People. Twenty-six of 28 public health nurse and allied health professional managers requested from regional managers were identified. There was no discipline manager available in psychology or social work in one region each. Interviews took place with 25 of the 26 disciplinary managers.

Regional health service managers provided information on responsibility for stroke services/co-ordination of provision of stroke services, service planning, local service provision and care pathways, while discipline-specific managers provided details on staffing levels, stroke management and service provision, and access to stroke services. Interview findings are presented under these headings.

### Responsibility for stroke services and co-ordination of provision of stroke services

Across the four health service regions, there was no designated co-ordinator or formal structured system for stroke service provision in any region. Regional managers were responsible for co-ordinating community services, but these were generic services, not specifically services for stroke. Provision of community services was generally influenced by the age and needs of the individual rather than being disease-specific, with those under age 65 years being managed by disability services and those aged 65 years and over managed by services for older people.

### Service planning

Across the four health service regions, there were no business plans for stroke care and no immediate intentions to develop such plans in any region. In general, business plans tended to be of a generic nature rather than specific to stroke.

When asked about plans for stroke services within the next two years, no specific stroke plans at regional level were reported. However, managers reported generic changes that would positively impact on stroke services – for instance developments in home-based therapy; developing and strengthening allied health professional services; and national roll-out of primary care teams. Two health service regions reported ongoing attempts to develop local stroke services.

### Local service provision and care pathways

Each regional manager reported several unmet needs in relation to current stroke service provision. These included inadequate staffing, significant variability in the availability of specialist staff both between and within regions, and the lack of age-appropriate services for stroke. Many community-based social workers, psychologists and speech and language therapists provided services to those aged 0–18 years only, because of legislative commitments in these areas. In addition, serious shortages of rehabilitation services for those under 65 years was noted in one region. Some stroke patients under the age of 65 had been placed in nursing homes, as there were no other suitable care options. Access to a national rehabilitation service for those under age 65 was described as limited due to long waiting lists. The discrepancy between acute care and continuing care was also highlighted nationally – lack of resources to ensure adequate transition from acute to community care was defined as a distinct unmet need. While there were generic care pathways, there were no clear care pathways in place for stroke care. Barriers were causing difficulties within existing care pathways for generic care and these in turn affected the delivery of stroke services. The barriers reported were unclear lines of communication between hospitals and primary care teams or multi-disciplinary teams (in certain areas such teams did not exist).

### Staffing levels

Considerable differences in community staffing levels were evident across disciplines and are summarized in Table 1. Information about staff involvement varied considerably across disciplines and regions. Approximate staff availability per health service region was calculated in order to provide some level of comparison across disciplines. For example, 7 of 32 regional areas reported 279 public health nurses. Estimated figures for one region were thus calculated as 319 (((279÷7) x 32 local health areas) ÷ 4 administrative healthcare regions). Table 1 shows relatively large numbers of public health nurses and physiotherapists available for stroke-related services; speech and language therapists and occupational therapists less common; while dieticians and psychologists were relatively rare, psychologists particularly so.

**Table 1:** Pattern of staffing of community-based health professionals nationally (Ireland)

	Estimated no. of staff posts per region* [N]	**Grades**			**Vacant posts% [N]**
		**Manager % [N]**	**Senior % [N]**	**Basic % [N]**	
Public health nursing [N: 7/32 LHO areas]	**319** [279.3]	4 [12]	22 [62]	71 [197^***^]	3 [8.5]
Physiotherapy [N:13/32 LHO areas]	**94** [153.6]	8 [12]	79 [121.6]	7 [20]	6 [10]
Occupational Therapy [N: 2 HSE regions]	**38** [77.0]	11 [8.4]	8 [63.2]	7 [5.5]	18 [13.9]
Social Work^**^ [N: all HSE regions]	**0.5** [2]	50 [1]	50 [1]	0 [0]	[N/A]
Dietetics [N: all HSE regions]	**20** [80.4]	11 [9]	81 [64.4]	8 [7]	9 [7.6]
Speech & Language Therapy [N: 27/32 LHO areas]	**67** [225.7]	11 [23.8]	62 [141.5]	27 [60.4]	13 [30]
Psychology [N: approx. 60% LHO areas]	**8** [20]	15 [3]	49 [10]	34 [7]	2 [0.5]

Bold figures represent estimated number per region.

* (wholetime equivalent per health care region).

* *These are social workers for adults – community based social work generic posts not involved in child protection.

No allied health professional was appointed to a hospital/community liaison role. Apart from one speech and language therapist in one area, no discipline had a specific discharge person for stroke, or dedicated community posts for stroke. Two regions had a total of three speech and language therapist posts designated for stroke - one based in a stroke rehabilitation unit. In other areas there were posts designated for adults, but not specifically stroke care.

The availability of community allied health professionals with specialist knowledge of stroke was queried. Two of the four geographic health service regions had a physiotherapist with specialist knowledge of stroke. In these regions, three physiotherapists were involved in therapy planning and served as a knowledge resource, but did not provide community-based services for patients. In two areas, there was an occupational therapist with specialist knowledge of stroke, one of whom was based in a specialist stroke rehabilitation unit, the other based in services for care of older people. In three of the four regions, there were identified lead speech and language therapists with specialist knowledge of stroke (1 to 2 per region), providing special clinics and long-term community support for stroke and contributing to therapy planning and service development.

The role of the dietetics service nationally was predominantly focussed on nutrition health promotion. There were no identified lead dieticians with specialist knowledge of stroke. There were no psychology posts designated for stroke, with the exception of an (unfilled) half-time post in a stroke rehabilitation unit. There were no psychologists nationally who acted as lead therapist or had specialist knowledge of stroke.

There were almost no social workers working in adult services nationally. In two regions, there was one social worker in community-based adult services (not specifically stroke-focussed). One worked with primary care teams and one with medical social work departments in hospitals. One social work manager had a liaison role between hospital and community, organising community care packages, including for those being discharged post-stroke.

Numbers of public health nurse staff were higher than other health professional disciplines. Generic only hospital/community liaison and discharge services were available in most areas. There were no designated public health nurse posts for stroke nationally, nor was there a lead stroke public health nurse in any region.

### Stroke patient numbers

No discipline was able to readily identify numbers of stroke patients in their region in the preceding year, some indicating that there was no way to obtain this data. With considerable effort by respondents, it was possible to get some indication in some regions. Very approximate numbers ranged from 205 to 700 per region, one region indicating that approximately 7 of every 1,300 patients in the region had stroke.

A stroke register did not exist in any region, nor did any discipline indicate that they maintained such a register within their own professional group. Some referred to the National Physical and Sensory Disability database, but this database did not include patients aged 65+ years. The absence of patient registers and multidisciplinary teams occurs in tandem with an uncentralised patient recording system which means that much information on patient needs and service provision is retained in a scattered manner, by differing professionals and services.

### Patient access

Patient access to community health professionals was variable (see Table 2 and Table 3). Access to public health nurse services was rated as good to excellent by respondents. Access was almost instant, with no waiting lists and services available to all people living at home. As public health nurse services were over-subscribed, the number of visits was likely to be limited. Patients tended not to be discharged from active care, but visits would be scaled down as need diminished.

**Table 2:** Access to services for stroke patients in the community^*^

	PHN^*^	PT^*^	OT^*^	SLT^*^	**Dietetics**	**Social work**	**Psychology**
**Access**	Very good	Reasonable	Reasonable-very limited	Very/quite limited	Absent-very limited	Absent-very limited	Absent-very limited
**Equal access for under 65 years/65+ years**	Very good	Very good	Limited(65+ mainly)	Very good	Absent (65+ only)	Absent (65+ only)	Absent (no service); ABI: under 65 only
**Limited duration of treatment**	No	In most cases	In many cases	In many cases	Absent(lack of service)	Absent (lack of service)	Absent (no service) ABI: Exception

^*^PHN = Public Health Nurse.

^*^PT = Physiotherapy.

^*^OT = Occupational therapy.

^*^SLT = Speech and language therapy.

**Table 3:** Community service availability

**Discipline**	**Waiting list?**		**Waiting time**	**Therapy duration**	**Long-term access to service**
	**Yes**	**No**			
PHN^*^		√	None	Based on need	Based on need
**Physiotherapy**	√		0–11 months	Typically 6–12 weeks	Limited
**Occupational therapy**	√		0–4 months	Variable (from 2 visits to service based on need/disability	Very limited
**Speech & Language**	√		Assessment: 1–3 months; Therapy: 3–9 months	Variable (from 6 weeks to service based on need)	Rarely
**Social work**	N/A		Service virtually unavailable	N/A	Rarely
**Dietetics**	N/A		Service virtually unavailable	N/A	No
**Psychology**	N/A		Service virtually unavailable	N/A	No

^*^PHN = Public Health Nurse.

Access to physiotherapy and occupational therapy services varied between limited and good. Where access was limited, patients under 65 years tended to be excluded. There were waiting lists for physiotherapy and occupational therapy. Access to social work, dietetics, psychological and speech and language therapy services was either limited or not available. Access to social work and dietetics was not available to patients under 65 years. While psychological services for stroke were non-existent, acquired brain injury (ABI) services were available in some areas, for those aged under 65 years only. There were no waiting lists for social work, dietetics or psychological services, because of the virtual non-existence of these services. Access to speech and language therapy, where available, was available to all ages. In 3 of the 4 regions there was a waiting list; on average 1–3 months for assessment and substantially longer (typically 6–9 months) for treatment. Duration of service provision from speech and language therapy also varied greatly.

Standardised outcome measures for stroke patients were used in some areas by some disciplines, but not routinely. For example, balance and mobility scales were used by physiotherapists, assessments before and after therapy by speech and language therapists, and cognitive and perceptual assessments by occupational therapists. There was no evidence of a consensus core dataset needed for all patients, nor of the availability or interpretability of results from standardised assessments from one discipline to another.

Where patients with stroke required long-term management, follow-up was described as frequently sporadic (Table 3). Resources and workload were cited as the primary reasons for being unable to guarantee long-term management. Limited interdisciplinary services and short-term respite care also prevented adequate long-term management of stroke. For a number of disciplines, review of patients with stroke was at the request of the public health nurse or another allied health professional, or through families or carers contacting the discipline directly. Formal review mechanisms were in place in only a minority of areas. Limitations to existing review procedures, where available, were the time needed to provide ongoing review to patients when new cases were coming on-stream all the time; the tendency to review ‘big’ cases, with the need to review ‘smaller’ cases not happening in many cases; lack of teams and standard protocols in the community; and waiting lists of people who have yet to receive a service, with no staff or resources to offer review services to existing clients. Ongoing referral from physiotherapy depended on the severity of the stroke and the patient’s potential for improvement. For most patients in receipt of speech and language therapy in the community, once the period of therapy ended, there was rarely any further contact with the service.

Out-of-hours support was not routinely available. Public health nurses could organise weekend essential services, but this was limited to priority patients, some of whom may have had a stroke. In some areas, ongoing support was in the form of a stroke group, or out-of-hours support was available in the form of providing respite for a number of hours to facilitate urgent family needs.

Once community rehabilitation ended, ongoing support for patients with stroke was variable. Patients might be referred to voluntary organisations or to a day hospital, day centre, or maintained at home with or without respite. There was a noted lack of day services nationally for patients with stroke under age 65, regardless of need. Access to specialist rehabilitation units was described as very limited, frequently involving long waiting times. If the family was in a position to manage, part of the ongoing care of the patient was likely to involve a home care package, if needed. In some areas stroke groups were organised, attended by patients, families and some allied health professionals. Some patients might continue to attend a day hospital, while others were referred to a specialist rehabilitation unit. However, while these services were available in some places, they were not available to many who needed them.

## Discussion

Findings of this study indicate that there was no dedicated, structured service for stroke in the community in Ireland and no immediate plans across health service regions to develop such a service. Community-based stroke rehabilitation and care was delivered through a generic service, in the same way as for any other condition. Stroke service provision was under the auspices of disability services or services for older people; the former provided services up to age 64 and the latter for those aged 65 years and over. The influence of age on service provision for stroke was not consistent, however, with some services more available to younger people (for example, occupational therapy and services for ABI), while others were only available to those aged 65+ years (for example, access to dietetic services and physiotherapy). For other services, such as public health nurse and speech and language therapy, there was little reported service differential based on age. The challenge was to provide a seamless service for patients with stroke in a community health service that was organised with a system (i.e., disability-related and (older) age-related services), which cross-cuts the needs for stroke, and other disease-specified groups. While the community service cannot adopt an indefinite list of specific and separate conditions on which to focus resource allocation, the question is whether the service should be managed to maximise the supports needed for a particular patient, regardless of diagnosis, or whether a specific sub-service, dedicated to stroke patients, should be the focus of developments.

In terms of overall provision of community services post-stroke, each healthcare region had a generic community rehabilitation service, sometimes provided in the context of a team, particularly where there was a primary care team in existence. However, this service did not always have access to all allied health professionals. Psychology was not represented, social work and dietetics were often not represented, while other disciplines such as speech and language therapy and occupational therapy were sometimes not represented. Community stroke rehabilitation teams were not available. The availability of an interdisciplinary care pathway for stroke varied greatly both between and within region, but was predominantly absent. This was true also of maintaining statistics about stroke patient populations in the community, which happened to some extent in a minority of local health areas and not at all in most.

Managers could not indicate the numbers of stroke patients in their local area/region. This absence of information on incidence and prevalence of stroke makes it difficult at strategy and planning levels to put in place the necessary funding and resources to develop and implement a comprehensive community-based stroke management service. The lack of central coordination of patient-related data in the community adds to the challenge of ensuring comprehensive patient care.

In relation to staffing, the numbers of public health nurses and physiotherapists potentially available for stroke-related services was relatively large, albeit in a context of significant current demand for these services across the health spectrum. Numbers of speech and language therapy and occupational therapy staff potentially available was considerably less. Dieticians, social workers and psychologists were relatively rare, or completely absent in some regions in the case of the latter two disciplines. The Bacon Report [19] identified significant deficiencies in the numbers of a range of community allied health professionals in Ireland. Increases of between 102% and 328% above existing numbers by 2015, depending on the discipline, were identified in order to meet the needs resulting from changing demographics and restructuring of health services.

It is acknowledged that stroke accounts for a higher proportion of healthcare spending than heart disease, due to its greater burden of disability (e.g., UK National Audit Office report, 2010 [20]). The greater part of these costs are incurred after the patient leaves hospital, as the disabling impact of stroke continues for the remainder of the person’s life. Maximising rehabilitation input helps to minimise the impact of disability. Early Supported Discharge (ESD) has been assessed in a number of studies internationally, described in a meta-analysis by Langhorne and colleagues [13]. International findings indicate that ESD services reduce long-term dependency and disability at 6 months for patients with mild to moderate stroke, also resulting in a stay, on average, of 8 days less in the acute hospital setting. A Cochrane Systematic Review [21] concluded that people after recent stroke were more independent and more likely to maintain abilities in relation to activities of daily living if they received therapy services at home. However, a more recent Cochrane review highlights the virtual absence internationally of evidence that investigates the efficacy of longer-term (one year after stroke) therapy-based rehabilitation interventions for patients with stroke [22].

Fragmentation of stroke services in the community is not an issue unique to Ireland [23]. It is a challenge currently being addressed in many health systems (for example, the Netherlands [24]; the US [25]; and the UK [20]). This fragmentation is likely to be exacerbated by regional variation in health service provision, an issue reported in the UK [26]. Considerable variation in service provision across Ireland was also noted in this survey, with as much variation within administrative healthcare regions as between them. This creates challenges for national service planning and evaluation. Standardisation of stroke services is one of the recommendations of the American Stroke Association task force on the development of stroke systems [25].

Study limitations included the use of a cascade approach to sampling of staff, as a result of which findings from the public health nurse and allied health professional manager surveys may not be representative. However, reports of managers within regions were consistent in terms of their reporting of stroke services regionally, and the consistency of these findings were sufficiently striking as to indicate significance and accuracy. In the absence of a developed and integrated service with quantifiable patient numbers and parameters, these findings can highlight the views of community health professionals of the diversity of community-level activity and of the variety of challenges experienced by staff in service delivery. The absence of patient registers meant it was not possible to quantify the extent of the service need and provision gap. However, staff views can highlight areas where services are inadequate, incomplete or absent.

## Conclusions

In conclusion, this study highlighted major gaps in the provision of community-based inter-disciplinary team services for people with stroke in one country. Where services existed, they were generic in nature, rarely inter-disciplinary in function and either deficient in (or completely deprived of) input from salient disciplines. Service managers and professionals identified a range of challenges to providing optimal care. These included the need for a strategic plan (which has since been addressed by a National Cardiovascular Health Policy [27]); increased funding including removal of healthcare staff employment ceilings; increased team resources and teamwork; and removal of service provision barriers based on age. Notably, there were many challenges beyond funding. These findings highlight the significant challenges to comprehensive rehabilitation and long-term management of patients with stroke that must be addressed in one healthcare system. Similar evaluations in other healthcare systems of community-based stroke health services are lacking. Such evaluations would serve to provide comparative lessons to serve to tackle this underserved aspect of care for patients with stroke and their families.

## Competing interests

The authors declare that they have no competing interests.

## Authors’ contributions

AH (community studies coordinator) contributed to the design of the study and method development, coordinated data collection and analysis, and drafted the manuscript; FH (hospital studies coordinator and overall project manager) contributed to the design of the study, to method development and study report writing, and helped to draft the manuscript; DON contributed to the design of the study and method development, and helped to draft the manuscript; HM contributed to the design of the study, to method development and study report writing, and helped to draft the manuscript. All authors read and approved the submitted manuscript.

## Pre-publication history

The pre-publication history for this paper can be accessed here:

http://www.biomedcentral.com/1472-6963/12/111/prepub
